# Pressure Masks for Facial Scar Treatment after Oncological Reconstruction: Long-Term Patient Satisfaction and Quality of Life

**DOI:** 10.1055/a-2035-4468

**Published:** 2023-04-03

**Authors:** Melissa De Henau, Sander M.J. van Kuijk, Carlo Colla, Eric Van den Kerckhove, Rene R.W.J. Van der Hulst, Andrzej Piatkowski

**Affiliations:** 1 Department of Plastic, Reconstructive and Hand Surgery, Maastricht University Medical Center, Maastricht, the Netherlands; 2 GROW School of Oncology and Reproduction, Maastricht University, the Netherlands; 3 Department of Clinical Epidemiology and Medical Technology Assessment (KEMTA), Maastricht University Medical Center, Maastricht, the Netherlands; 4 Department of Rehabilitation Sciences, Faber, Universitaire Ziekenhuizen Leuven, Leuven, Belgium; 5 Department of Physical Medicine and Rehabilitation and Burns Center, Universitaire Ziekenhuizen Leuven, Leuven, Belgium; 6 Department of Plastic Surgery, VieCuri Medical Centrum, Venlo, the Netherlands

**Keywords:** scars, facial pressure mask, pressure therapy, quality of life, patient-reported outcome

## Abstract

**Introduction** With increasing incidence of facial skin cancer, more patients undergo facial reconstruction following Mohs micrographic surgery (MMS). Aesthetically unpleasing, thickened facial flaps, and disturbing scars can be treated with a pressure mask with inner silicone lining to help improve functional and aesthetic outcomes. However, data on long-term patient satisfaction and quality of life (QoL) following this treatment are lacking.

**Methods** We aimed to assess long-term satisfaction and QoL of patients who underwent local flap reconstruction following MMS. Patients treated between January 2012 and October 2020 were invited to answer FACE-Q and SCAR-Q questionnaires. Demographic data, skin cancer type and location, type of reconstruction, postoperative complications, duration of pressure mask therapy, daily compliance, and additional scar treatment were collected to explore possible predictors.

**Results** Of 92 eligible patients, 50 responded. Eighteen respondents were male (36%) and 32 were female (64%). Mean duration of pressure mask therapy was 10.20 ± 4.61 months. Patients were 61.14 ± 32.91 months after completion of pressure mask therapy upon participation. Patients whose reconstruction consisted of multiple flaps had significantly worse outcomes in social function ( *p* = 0.012), scar appearance ( *p* = 0.045), and scar symptoms ( *p* = 0.008). A trend of increasing time since therapy completion predicting better outcomes was observed for all scales, and it was a significant predictor for better scar appearance ( *p* = 0.001) and less scar symptoms ( *p* = 0.001).

**Conclusion** Pressure mask treatment for facial flaps and scars following MMS results in good long-term patient satisfaction and QoL. Multiple local flaps, reflecting a larger skin defect postexcision, is a predictor for worse outcomes in social function, scar appearance, and symptoms. Increasing time is associated with increasing satisfaction, which reflects satisfactory and stable long-term effects of treatment, possibly combined with more acceptance of the result over time.

## Background

Most skin cancers are located in the head and neck area, and overall incidence is still increasing. 1 2 3 Mohs micrographic surgery (MMS) is currently widely used to excise skin tumors, while attempting to maximally preserve healthy tissue. 4 Reconstruction of defects following MMS can be challenging, given the functional and cosmetic demands in the head and neck area. 5 Moreover, postsurgical facial scars can cause severe psychological, emotional, and social burden for patients. 6 7 When local flaps are used, the postsurgical scars extend from the original defect to the donor site. Reoperations are often necessary following reconstruction to further improve aesthetic and functional outcome. 8 In some cases, facial scars contract or become hypertrophic, and some flaps need further surgical thinning. In our center, extensive local flaps following facial MMS are treated with a facial pressure mask with inner silicone liner. This treatment has been shown to be effective as an adjuvant therapy for unsatisfactory aesthetic results after facial flap surgery since it reduces flap thickness and scar erythema, and improves pliability of the skin and scar. 9 However, data on long-term efficacy, patient-reported satisfaction, and quality of life (QoL) after facial pressure mask therapy following local flap reconstruction are limited. Therefore, we aimed to assess patient satisfaction, QoL, and patient-reported long-term efficacy of facial pressure mask therapy. Secondarily, we aimed to explore possible predictors of QoL and patient satisfaction.

## Methods

### Setting and Study Population

For this cohort study, patients who received pressure mask treatment following local flap reconstruction for facial MMS defects were recruited from the department of plastic and reconstructive surgery in an academic setting. An experienced orthotist/prosthetist manually fabricated these masks as described previously by Colla et al. 10 All adult patients (e.g., older than 18 years of age) who started facial pressure mask therapy between January 2012 and October 2020 to treat scar and flap irregularities were invited for participation. Patients with a follow-up of less than 3 months and patients who were still undergoing pressure mask therapy were excluded. Another exclusion criterion was not being able to fill in the questionnaires (e.g., non-Dutch speakers and cognitively impaired patients).

The study conformed to good clinical practice guidelines and followed the recommendations of the Declaration of Helsinki. The protocol was approved by the local ethics committee (METC 2021-2798).

### Data Collection

After providing informed consent, participants were asked to complete four different FACE-Q questionnaires and the SCAR-Q questionnaire. Questionnaires were collected between October 2021 and January 2022. The FACE-Q is a validated patient-reported outcome measure (PROM) developed to evaluate the experience and outcomes of aesthetic facial procedures from a patient's perspective. 11 12 13 The framework for FACE-Q Aesthetics covers three domains: appearance, health-related quality of life (HRQOL), and adverse effects. Each domain is composed of multiple independently functioning scales. The following scales were used in this study: satisfaction with overall facial appearance (appearance domain), satisfaction with result (HRQOL), satisfaction with decision (HRQOL), and social function (HRQOL). The SCAR-Q is another validated PROM and consists of three scales that measure scar appearance, scar symptoms, and psychosocial impact. 14 15

The participants were instructed to fill in the questionnaires based on their current state of satisfaction and functioning. Responses to the questionnaires were scored on a Likert scale ranging from 1 to 4. For each scale, the total score was calculated and standardized to a Rasch-transformed score ranging from 0 to 100. Higher Rasch-transformed scores represent a greater patient satisfaction or QoL.

Other variables that we collected were age, sex, type of skin cancer, location and type of reconstruction, postoperative complications, duration of pressure mask therapy, the average daily amount of hours that patients wore the mask (< 8, 8–12, or 12–16 hours), time since completion of pressure mask therapy, and additional scar treatments following pressure mask therapy. Additional scar treatments were defined as conservative treatment (hydrating creams, skin therapy, additional silicone gels, or sheets following pressure mask therapy), surgery, and a combination of surgery with conservative treatment.

### Data Analysis

Data on continuous variables is displayed as mean ± standard deviation. Data on categorical variables is presented as frequencies and percentages. Simple and multiple linear regression analysis have been performed to assess a possible association between the independent variables and the outcomes. The following variables were assessed as possible predictors: age, sex, skin cancer type (basal cell carcinoma or other), location of defect, type of reconstruction, duration of pressure mask therapy, daily compliance, time since therapy completion, and additional treatment. Continuous data on duration of pressure mask therapy and time since therapy completion was used for regression analysis. Regression coefficients with the corresponding 95% confidence intervals (CI) are provided. A value of *p* < 0.05 was considered statistically significant. Statistical analyses were performed using statistical software program SPSS 25.0 (IBM, Armonk, NY).

## Results

Of the 92 eligible patients who were invited for participation, 50 responded and filled out the questionnaire (54.35% response rate). The male-to-female ratio of the respondents was 36 to 64%. Population characteristics are presented in Table 1 . Mean duration of pressure mask therapy was 10.20 ± 4.61 months. Patients were 61.14 ± 32.91 months after completion of the pressure mask therapy when filling in the questionnaire (minimum 3 months, maximum 111 months).

**Table 1:** Sample characteristics

Age	Mean	SD	Postoperative complication	*N*	%
Male	65.2	13.5	None	43	86
Female	67.0	9.8	Minor bleeding	5	10
Overall	66.4	11.1	Dehiscence	2	4
**Cancer type**	***N***	**%**	**Therapy duration**		
BCC	33	66	< 6 mo	12	24
SCC	4	8	6–12 mo	23	46
Lentigo maligna	5	10	> 12 mo	15	30
Melanoma	5	10	**Daily compliance**		
Keratoacanthoma	1	2	< 8 h per day	13	26
EMPSGC	1	2	8–12 h per day	29	58
Not reported	1	2	> 12 h per day	8	16
**Location of defect**			**Time since therapy completion**		
Nose	27	54	< 2 y	9	18
Cheek	14	28	2–4 y	11	22
Lip	2	4	4–6 y	8	16
Medial canthus	4	8	6–8 y	13	26
Multiple locations	3	6	> 8 y	9	18
**Type of reconstruction**			**Additional treatment**		
Forehead flap	18	36	None	19	38
FTG	5	10	Conservative	23	46
Advancement/Rotation	17	34	Surgical	5	10
Multiple	8	16	Both	3	6
Not reported	2	4			

Abbreviations: BCC, basal cell carcinoma; EMPSGC, endocrine mucin-producing sweat gland carcinoma; FTG, full-thickness skin graft; SCC, squamous cell carcinoma; SD, standard deviation.

One patient underwent additional surgery after facial pressure mask therapy because of tumor recurrence, whereas four received further surgical scar and/or flap correction.

**Fig. 1:**
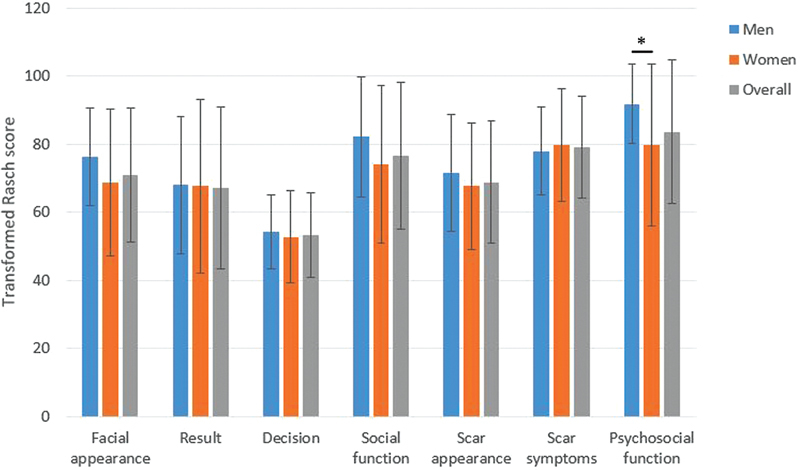
Mean FACE-Q scores for satisfaction with facial appearance, satisfaction with result and decision, and social function, and the items of scar appearance, scar symptoms, and psychosocial function of the SCAR-Q. Blue bars indicate results for men, orange bars indicate results for women and the gray bars indicate overall mean. The whiskers represent standard deviation. * indicates a statistically significant difference ( *p* < 0.05).

FACE-Q scores for satisfaction with facial appearance, satisfaction with the result and decision, and social function did not significantly differ between men and women, whereas SCAR-Q psychosocial function was significantly lower for women ( *p* = 0.038) ( Fig. 1 and Table 2 ). Regression coefficients of linear regression analysis for FACE-Q outcomes are presented in Table 2 , whereas regression coefficients of linear regression analysis for SCAR-Q outcomes can be found in Table 3 .

**Table 2:** Regression coefficients of linear regression analysis for FACE-Q outcomes

	Satisfaction with appearance		Satisfaction with result		Satisfaction with decision		Social function	
Characteristic	Regression coefficient (95% CI)	*p* -Value	Regression coefficient (95% CI)	*p* -Value	Regression coefficient (95% CI)	*p* -Value	Regression coefficient (95% CI)	*p* -Value
Sex								
Male	0 [Reference]		0 [Reference]		0 [Reference]		0 [Reference]	
Female	–8.43 (–19.91 to 3.04)	0.146	–1.26 (15.23–12.91)	0.859	–1.45 (–8.99 to 6.09)	0.701	–8.64 (–21.23 to 3.95)	0.174
Age in years	–0.20 (–0.71 to 0.31)	0.430	–0.25 (–0.87 to 0.36)	0.409	–0.03 (–0.35 to 0.30)	0.872	0.17 (–0.38 to 0.73)	0.529
Cancer type								
BCC	0 [Reference]		0 [Reference]		0 [Reference]		0 [Reference]	
Other	–0.12 (–12.35 to 12.11)	0.984	–2.38 (–17.17 to 12.42)	0.748	–1.97 (–9.71 to 5.77)	0.611	–1.01 (–14.42 to 12.41)	0.881
Location of defect								
Nose	0 [Reference]		0 [Reference]		0 [Reference]		0 [Reference]	
Cheek	0.36 (–12.84 to 13.55)	0.957	4.74 (–11.30 to 20.78)	0.555	1.62 (–6.85 to 10.09)	0.703	–11.65 (–25.22 to 1.92)	0.091
Lip	8.00 (–21.36 to 37.36)	0.586	–16.26 (–51.96 to 19.44)	0.364	–12.39 (–31.13 to 6.36)	0.190	0.35 (–29.84 to 30.54)	0.981
Medial canthus	–13.00 (–34.46 to 8.46)	0.229	–4.51 (–30.61 to 21.59)	0.729	–3.89 (–17.61 to 9.84)	0.571	–24.90 (–46.97 to –2.83)	0.028 a
Multiple	8.33 (–16.05 to 32.71)	0.495	9.41 (–20.24 to 39.05)	0.526	4.62 (–10.96 to 20.19)	0.554	11.19 (–13.89 to 36.26)	0.374
Type of reconstruction								
Forehead flap	0 [Reference]		0 [Reference]		0 [Reference]		0 [Reference]	
FTG	3.45 (–16.60 to 23.50)	0.731	–5.55 (–29.60 to 18.50)	0.644	–3.70 (–16.37 to 8.97)	0.559	–2.60 (–23.19 to 17.99)	0.800
Advancement/Rotation	–1.96 (–15.19 to 11.27)	0.767	–6.44 (–22.31 to 9.42)	0.418	–2.17 (–10.53 to 6.19)	0.604	–11.75 (–25.34 to 1.83)	0.088
Multiple	–8.425 (–25.20 to 8.35)	0.317	–14.15 (–34.27 to 5.97)	0.164	–8.84 (–19.97 to 2.29)	0.116	–22.28 (–39.50 to –5.05)	0.012 a
Postoperative complications								
No	0 [Reference]		0 [Reference]		0 [Reference]		0 [Reference]	
Yes	–3.45 (–19.55 to 12.85)	0.679	–1.82 (–21.42 to 17.78)	0.852	2.64 (–7.73 to 13.02)	0.611	0.49 (–17.27 to 18.25)	0.956
Duration of pressure therapy (in months)	0.29 (–0.94 to 1.52)	0.639	–0.301 (–1.79 to 1.19)	0.686	–0.22 (–1.02 to 0.57)	0.574	0.64 (–0.69 to 1.98)	0.338
Daily compliance								
< 8 h	0 [Reference]		0 [Reference]		0 [Reference]		0 [Reference]	
8–12 h	–5.06 (–18.34 to 8.23)	0.448	0.85 (–15.04 to 16.74)	0.915	–1.51 (–10.25 to 7.23)	0.729	–4.47 (–18.83 to 9.89)	0.534
> 12 h	1.71 (–16.18 to 19.60)	0.848	13.66 (–7.73 to 35.06)	0.205	3.79 (–7.83 to 15.41)	0.515	8.28 (–11.05 to 27.61)	0.393
Time since therapy completion (in months)	0.03 (–0.15 to 0.20)	0.745	0.14 (–0.06 to 0.35)	0.164	0.10 (–0.01 to 0.21)	0.067	0.13 (–0.06 to 0.316)	0.163
Additional therapy								
None	0 [Reference]		0 [Reference]		0 [Reference]		0 [Reference]	
Conservative	–8.80 (–20.99 to 3.83)	0.153	–5.36 (–20.42 to 9.70)	0.477	–6.11 (–14.04 to 1.81)	0.127	–11.69 (–24.86 to 1.48)	0.081
Surgery	3.82 (–15.93 to 23.59)	0.390	0.88 (–23.53 to 25.30)	0.942	0.24 (–12.47 to 12.96)	0.970	4.39 (–16.96 to 25.74)	0.681
Conservative + surgery	2.97 (–21.46 to 27.39)	0.244	–13.32 (–53.49 to 16.86)	0.379	–2.16 (–17.88 to 13.56)	0.783	–11.54 (–37.94 to14.85)	0.383

Abbreviations: BCC, basal cell carcinoma; CI, confidence interval; FTG, full-thickness skin graft.

a Statistically significant result ( *p* < 0.05).

**Table 3:** Regression coefficients of linear regression analysis for SCAR-Q outcomes

	Scar appearance		Scar symptoms		Psychosocial function	
Characteristic	Regression coefficient (95% CI)	*p* -Value	Regression coefficient (95% CI)	*p* -Value	Regression coefficient (95% CI)	*p* -Value
Sex						
Male	0 [Reference]		0 [Reference]		0 [Reference]	
Female	–4.21 (–14.86 to 6.44)	0.431	1.72 (–7.18 to 10.62)	0.700	–12.80 (–24.83 to –0.77)	0.038 a
Age in years	–0.08 (–0.54 to 0.39)	0.744	0.02 (-0.37–0.41)	0.921	–0.10 (–0.65 to 0.44)	0.703
Cancer type						
BCC	0 [Reference]		0 [Reference]		0 [Reference]	
Other	–1.65 (–12.77 to 9.46)	0.766	–2.01 (–11.31 to 7.29)	0.666	–0.29 (–13.45 to 12.87)	0.965
Location of defect						
Nose	0 [Reference]		0 [Reference]		0 [Reference]	
Cheek	–7.10 (–19.09 to 4.90)	0.240	–15.21 (–24.31 to –6.10)	0.002 a	–6.39 (–20.57 to 7.78)	0.368
Lip	5.26 (–21.43 to 31.95)	0.693	–14.85 (–35.11 to 5.41)	0.147	14.96 (–16.58 to 46.51)	0.344
Medial canthus	6.01 (–13.50 to 25.52)	0.538	–9.35 (–24.16 to 5.46)	0.210	–6.04 (–29.10 to 17.02)	0.601
Multiple	6.93 (–15.24 to 29.09)	0.532	–2.52 (–19.34 to 14.31)	0.764	4.63 (–21.57 to 30.83)	0.724
Type of reconstruction						
Forehead flap	0 [Reference]		0 [Reference]		0 [Reference]	
FTG	–1.35 (–18.96 to 16.26)	0.878	–7.60 (–21.72 to 6.52)	0.284	–1.05 (–22.64 to 20.54)	0.922
Advancement/Rotation	1.34 (–10.28 to 12.96)	0.817	–0.37 (–9.68 to 8.95)	0.938	2.48 (–11.77 to 16.73)	0.728
Multiple	–15.08 (–29.81 to –0.34)	0.045 a	–16.35 (–28.16 to –4.54)	0.008 a	–7.18 (–25.24 to 10.89)	0.428
Post-operative complications						
No	0 [Reference]		0 [Reference]		0 [Reference]	
Yes	–2.83 (–17.34 to 11.98)	0.703	–3.61 (–15.89 to 8.68)	0.558	3.08 (–14.32 to 20.47)	0.724
Duration of pressure therapy (in months)	–0.57 (–1.69 to 0.54)	0.308	–0.54 (–1.47 to 0.38)	0.244	0.15 (–1.17 to 1.47)	0.820
Daily compliance						
< 8 h	0 [Reference]		0 [Reference]		0 [Reference]	
8–12 h	–4.02 (–15.91 to 7.87)	0.500	–0.33 (–9.99 to 9.32)	0.945	–10.46 (–24.51 to 3.58)	0.141
> 12 h	8.40 (–7.61 to 24.41)	0.296	12.89 (–0.10 to 25.89)	0.052	–2.78 (–21.69 to 16.13)	0.769
Time since therapy completion (in months)	0.20 (0.05 to 0.35)	0.008 a	0.20 (0.09 to 0.32)	0.001 a	0.18 (–0.002 to 0.35)	0.053
Additional therapy						
None	0 [Reference]		0 [Reference]		0 [Reference]	
Conservative	–7.55 (–18.52 to 3.42)	0.172	–4.37 (–13.40 to 4.66)	0.335	–9.81 (–23.01 to 3.38)	0.141
Surgery	7.38 (–10.40 to 25.16)	0.408	5.57 (–9.07 to 20.21)	0.448	–0.42 (–21.82 to 20.97)	0.969
Conservative + surgery	–13.75 (–35.73 to 8.22)	0.214	–17.97 (–36.06 to 0.13)	0.052	–3.75 (–30.20 to 22.69)	0.776

Abbreviations: BCC, basal cell carcinoma; CI, confidence interval; FTG, full-thickness skin graft.

a Statistically significant result ( *p* < 0.05).

### Satisfaction with Appearance of the Face

Linear regression analysis for satisfaction with facial appearance did not identify statistically significant predictors. Patient satisfaction seemed to increase with increasing therapy duration (0.29 per month, *p* = 0.639) and increasing time since therapy completion (0.03 per month, *p* = 0.745).

### Satisfaction with the Result

No statistically significant predictors were found for satisfaction with result. The type of reconstruction seemed to, although statistically not significant, influence the satisfaction with the result; in a multiple linear model with forehead flaps as a baseline, multiple local flap techniques were associated with lower scores ( *p* = 0.164). Linear regression analysis assessing daily compliance indicated that satisfaction seemed to be higher in patients who wore the mask for more than 12 hours per day. Mean satisfaction with result was 64.46 (95% CI [50.38–78.54]), 65.31 (95% CI [56.36–74.26]), and 78.13 (95% CI [57.38–98.87]) with a daily compliance of less than 8 hours, 8 to 12 hours, and more than 12 hours, respectively. Simple linear regression for time since completion of therapy indicated a positive linear association, however, not statistically significant (0.14 per month, *p* = 0.164).

### Satisfaction with Decision

It should be noted that for this scale, since health care insurance covered the costs of treatment, patients could not answer one question, and therefore the maximum Rasch score is lower than for the other scales used in this study. Even though not statistically significant, multiple flaps showed a potentially clinically meaningful association with lower satisfaction with the decision ( *p* = 0.116). Satisfaction was positively associated with time since therapy completion (in months) in simple linear regression (0.10 per month, *p* = 0.067).

### Social Function

In a multiple linear regression model with nose as a baseline for defect location, having a defect on medial canthus was a statistically significant predictor for a worse outcome ( *p* = 0.028). On average, patients with a defect on the medical canthus (mean 56.25, 95% CI [45.51–66.99]) scored 24.90 points lower than patients whose defect was located on the nose (mean 81.15, 95% CI [74.06–88.23]). Furthermore, reconstruction by multiple flaps was a significant predictor for worse social function in a multiple linear model with forehead flaps as baseline ( *p* = 0.012).

### Satisfaction with Scar Appearance

Patients reported significantly lower satisfaction with scar appearance when they underwent reconstruction with multiple local flaps ( *p* = 0.045). Satisfaction with scar appearance increased with time since therapy completion (0.20 per month, *p* = 0.008).

### Burden of Scar Symptoms

**Fig. 2:**
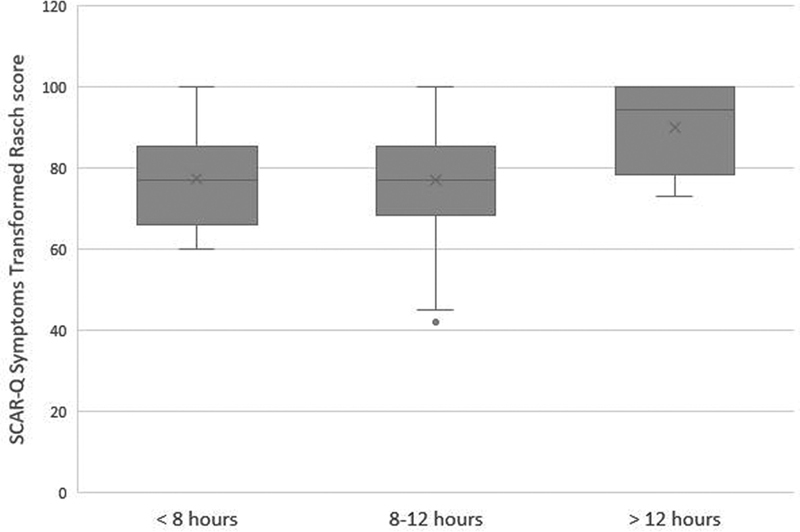
SCAR-Q symptoms transformed Rasch score per category of daily compliance. Higher SCAR-Q scores correspond to less burden of scar symptoms and greater satisfaction.

Predictors associated with a worse burden of scar symptoms were skin cancers located on the cheek ( *p* = 0.002) and reconstructions with multiple local flaps ( *p* = 0.008). Lower burden of scar symptoms was observed with increasing time since therapy completion (0.20 per month, *p* = 0.001). A trend toward lower burden of scar symptoms was observed with a daily compliance of > 12 hours per day ( *p* = 0.052), as illustrated in Fig. 2 .

### Psychosocial Functioning

Sex was a predictor for psychosocial functioning in this cohort, with a significantly worse outcome for women ( *p* = 0.038); women scored on average 12.80 points lower than men. Time since therapy completion predicted psychosocial function in a simple linear regression model ( *R* ^2^ = 0.076; regression coefficient 0.18, *p* = 0.053), and in a multiple linear regression model with sex ( *R* ^2^ of model = 0.174, *p* = 0.011; regression coefficient of gender –13.63, *p* = 0.022, regression coefficient of time since completion 0.89, *p* = 0.031).

## Discussion

In this study, we assessed long-term satisfaction and QoL of patients who were treated with facial pressure mask therapy following local flap reconstruction of MMS facial skin defects. Our data confirm long-term satisfactory results of facial pressure mask therapy over a period of approximately 9 years. The proportion of male and female participants, and the rates of skin types are in accordance with previous studies, indicating that our sample is representative in terms of sex and cancer type distribution. 2 8

The burden for patients of wearing a full-face pressure mask should not be underestimated. In this setting of postsurgical flaps and scars, patients were instructed to wear the mask for a minimum of 8 to 12 hours per day. Satisfactory functional results with this regimen have already been reported. 9 16

We deliberately chose the scales of the FACE-Q aesthetic modules as outcome measures for patients that were treated for skin cancer. Since we were interested in long-term aesthetic satisfaction, the aesthetic module was deemed more appropriate and relevant. When comparing outcomes for facial appearance from our study to outcomes following aesthetic procedures, we conclude that patients who underwent facial pressure mask therapy following local flap reconstruction were equally or more satisfied with their facial appearance. 17 18 Compared to nonsurgical aesthetic interventions (e.g., Botox, filler, skin treatments), patients from our population report higher satisfaction with facial appearance, whereas surgical aesthetic interventions (rhinoplasty, facelift, blepharoplasty) lead to similar satisfaction scores. 17 Satisfaction with facial appearance following nanofat injection in depressed facial scars were very similar to the rates reported in our study. 18 In general, patients seeking aesthetic improvements often have higher burden of self-consciousness of appearance and hold certain expectations toward the desired outcome. 19 This may reflect their state of satisfaction after the procedure, which is slightly lower than in the reconstructive setting. This hypothesis is supported by the findings of Elegbede et al, who reported rates of satisfaction with facial appearance following facial trauma reconstruction that were similar to the rates found in our study. 20 Despite the population being predominantly male and younger (mid-twenties to mid-forties) in this latter study, satisfaction with facial appearance and satisfaction with the result lie within the same range as reported in our study. However, our patients display slightly higher scores in social function.

Our study did not identify significant predictors for satisfaction with facial appearance, satisfaction with the result, and satisfaction with the decision. However, previous research has shown that a daily adherence of more than 12 hours is associated with better outcomes regarding satisfaction with the result compared to an adherence of less than 8 hours per day. 16 Similarly, satisfaction with the decision of wearing the facial pressure mask was significantly greater in patients who wore the mask 8 to 12 hours and 12 to 16 hours per day than in patients who wore the mask less than 8 hours per day. Although not statistically significant, a trend toward greater satisfaction with facial appearance and less scars symptoms was also observed in this study.

For all scales, time since therapy completion was positively associated with better outcomes, and was a statistically significant predictor of scar appearance, scar symptoms, and psychosocial functioning. This is in accordance with previous findings, where the severity of the body image disturbance decreased over time in patients who recovered from head and neck cancer. 21 In another study, no significant difference in satisfaction with the result, decision, and social function over time was observed. 16 However, in this study patients with different indications were included (following MMS, trauma, and burns). Our findings of increased satisfaction and QoL over time could be due to the possibility that it might take the patients several years to accept facial irregularities and process the diagnosis and treatment following skin cancer. Hypothetically, patients might be less occupied by their disease and facial appearance as more time goes by. Another possible explanation could be due to a prolonged maturation period of the scar and flap tissue, which has been reported to last up to 2 years or more. 22 However, it seems unlikely that scar maturation lasts up to 8 years, indicating that the latter cannot be the sole explanation to our observation of better outcomes with increased time since treatment.

Another factor that predicted outcomes was the use of multiple flaps for reconstruction. The use of multiple flaps was significantly associated with worse outcomes in social function, scar appearance, and scar symptoms. A trend toward decreased satisfaction with facial appearance, result, and decision was also observed. The necessity to use multiple flaps clinically indicates a larger defect following MMS. Logically, larger defects require more mobilization of surrounding tissue, and are most likely associated with more donor site morbidity. When comparing different reconstructive approaches, larger defects and more complex reconstructions predict worse patient-reported satisfaction and QoL. 21 23 24 Furthermore, scars located on the cheek were also associated with worse scores on the scar symptoms scale. This might be due to the mobility of the cheek during mastication, speech, and facial expression, giving rise to a more tense sensation of scars in that area.

Sex was only a significant predictor for psychosocial function, where female patients scored significantly lower than male patients. Similarly, Ziolkowski et al identified sex as a predictor for worse psychosocial outcomes. 25 In contrast, Miranda et al found better outcomes in women. 26 The latter study assessed patients following scar revision surgery after both traumatic and elective scars and found that women more frequently reported improvements than men after the revision of elective scars. 26 Thus, the notion of better outcomes in women refers to improvements following scar revision surgery rather than in the setting prior to revision. The finding of differences in psychosocial functioning between sexes could be due to possible personality differences between females and males, regarding the cosmetic appearance of scars, combined with higher expectations, which could negatively influence the patient's opinion of their scar. In our population, we hypothesize that the impact of having a facial scar weighs more on women's psychosocial well-being compared to men.

## Strengths and Limitations

To our knowledge, this is the first study to combine both aesthetic FACE-Q modules with the recently developed and validated SCAR-Q questionnaire. This allows assessment of patient-reported satisfaction on both the aesthetic aspects of the reconstruction and functional repercussions, as well as evaluation of the face and the scar. This study provides new insight on the long term (up to 9 years) after facial pressure mask therapy for flaps and scars post-MMS. The strength of this study is the long follow-up period. In general, the long-term results are reported with a 1- to 5-year follow-up, while we provide data on a follow-up period of up to 9 years. However, this study has several limitations. The sample size, although representative in terms of cancer type and gender distribution, is rather small. This might be a source of bias, since it could be possible that the most dissatisfied or most satisfied patients participated. Another limitation to this study is the variability of reported outcomes between patients. This combined with a relatively small sample size, impedes the power to find statistically significant differences. Although we found some trends, statistical significance was not reached on some items.

Additionally, this study focuses on one cohort of patients and does not have a control group. With retrospective designs, it can be challenging to create a control group of matching patients, especially in this setting of specialized treatment of more extensive facial flaps and scars. However, ideally, future studies should attempt to include a control group to further investigate the effects of this therapy compared to controls and other treatment options.

Furthermore, patients were instructed to wear the pressure mask as long as they could endure, preferably more than 8 hours each day; however, this data about therapy compliance is reported by patients and could not be objectified. Therefore, these data might be susceptible for bias. Regarding the finding of worse predictive outcomes with reconstructions by multiple flaps, we hypothesized this was due to larger and/or more complex skin defects. Despite this logical assumption, for future research, defect size should be measured as a variable to assess whether or not this assumption is truly correct.

## Conclusion

This cohort study illustrates overall satisfactory long-term aesthetic results and QoL following facial reconstruction and pressure mask therapy. Satisfaction and QoL improved over time, which might also reflect patients' acceptance with their appearance and scars, as well as good long-term efficacy of treatment. The need for multiple local flaps for reconstruction, which most likely reflects moderately larger skin defects, is a predictor for worse outcomes regarding social function, scar appearance, and scar symptoms. This study can help to better inform patients about possible outcomes after facial pressure mask therapy for local flap reconstruction, and help to manage patient expectations.
